# Psychiatric adverse events following COVID-19 vaccination: a population-based cohort study in Seoul, South Korea

**DOI:** 10.1038/s41380-024-02627-0

**Published:** 2024-06-04

**Authors:** Hong Jin Kim, Min-Ho Kim, Myeong Geun Choi, Eun Mi Chun

**Affiliations:** 1 Department of Orthopedic Surgery, Inje University Sanggye Paik Hospital, College of Medicine, Inje University, Seoul, Republic of Korea; 2 Informatization Department, Ewha Womans University Seoul Hospital, Seoul, Republic of Korea; 3 Division of Pulmonology and Critical Care Medicine, Department of Internal Medicine, School of Medicine, Ewha Womans University, Seoul, Republic of Korea

**Keywords:** Depression, Bipolar disorder, Schizophrenia

## Abstract

Evidence has suggested an increased risk of psychiatric manifestations following viral infections including coronavirus disease-2019 (COVID-19). However, psychiatric adverse events (AEs) after COVID-19 vaccination, which were documented in case reports and case series, remain unclear. This study is aimed to investigate the psychiatric AEs after COVID-19 vaccination from a large population-based cohort in Seoul, South Korea. We recruited 50% of the Seoul-resident population randomly selected from the Korean National Health Insurance Service (KNHIS) claims database on 1, January, 2021. The included participants (*n* = 2,027,353) from the Korean National Health Insurance Service claims database were divided into two groups according to COVID-19 vaccination. The cumulative incidences per 10,000 of psychiatric AEs were assessed on one week, two weeks, one month, and three months after COVID-19 vaccination. Hazard ratios (HRs) and 95% Confidence interval (CIs) of psychiatric AEs were measured for the vaccinated population. The cumulative incidence of depression, anxiety, dissociative, stress-related, and somatoform disorders, sleep disorders, and sexual disorders at three months following COVID-19 vaccination were higher in the vaccination group than no vaccination group. However, schizophrenia and bipolar disorders showed lower cumulative incidence in the vaccination group than in the non-vaccinated group. Depression (HR [95% CI] = 1.683 [1.520–1.863]), anxiety, dissociative, stress-related, and somatoform disorders (HR [95% CI] = 1.439 [1.322–1.568]), and sleep disorders (HR [95% CI] = 1.934 [1.738–2.152]) showed increased risks after COVID-19 vaccination, whereas the risks of schizophrenia (HR [95% CI] = 0.231 [0.164–0.326]) and bipolar disorder (HR [95% CI] = 0.672 [0.470–0.962]). COVID-19 vaccination increased the risks of depression, anxiety, dissociative, stress-related, and somatoform disorders, and sleep disorders while reducing the risk of schizophrenia and bipolar disorder. Therefore, special cautions are necessary for administering additional COVID-19 vaccinations to populations vulnerable to psychiatric AEs.

## Introduction

In the unprecedented era of coronavirus disease-2019 (COVID-19), the global outbreak of COVID-19 has had an unpredictable and heterogeneous impact on the healthcare system worldwide [1, 2]. Especially in mental illness, COVID-19 showed an increased risk of mental health problems together with lockdown, social distancing, and uncertain causes [3, 4]. There have been growing concerns that the COVID-19 pandemic has increasingly had a detrimental effect on long-term mental health at an early stage in the development of vaccines [5, 6].

The rapid development of COVID-19 vaccines, ranging from mRNA-based vaccines (BNT162b2, mRNA-1273) to viral vector vaccines (cDNA-based vaccines; AZD1222, JNJ-78436735), has contributed to overcoming the COVID-19 pandemic in the view of severity and mortality [1, 7]. However, it has also given rise to new issues such as post-COVID-19 sequelae and vaccine-related adverse events (AEs) [2, 5–10]. With their issues, mental health is still an unsolved concern in the post-COVID-19 era [4, 6]. Many studies have focused on the correlation between mental health and COVID-19 breakthrough [11]. However, mental illness as a result of the COVID-19 vaccine itself, specifically post-vaccination psychiatric AEs was not well-studied, with scant evidence in the literature, which was documented primarily in the form of case reports and case series [12–15].

In this study, we investigated the psychiatric AEs including schizophrenia, depression, bipolar disorder, anxiety, dissociative, stress-related, and somatoform disorders, sleep disorders, and sexual disorders after COVID-19 vaccination from a population-based cohort using the Korean National Health Insurance Service (KNHIS) claims database in Seoul, South Korea.

## Materials and methods

The concept and protocol of this study were approved by the Institutional Review Board (IRB) of our institute, which waived the requirement for informed consent because data analyses were performed retrospectively using anonymized data derived from the South Korean NHIS database.

### Data source

We used the KNHIS claims database to recruit a randomly selected 50% of the population residing in Seoul on 1 January 2021 with their diagnostic records up to 31 December 2021. The process of selecting a random 50% of the population in Seoul was carried out by the KNHIS system. After authorization by KNHIS, the data collection was performed in November 2022. The psychiatric AEs included schizophrenia, mood disorders (depression, and bipolar disorder), anxiety, dissociative, stress-related, and somatoform disorders (anxiety disorders, obsessive-compulsive disorder, reacting to severe stress, and adjustment disorders, conversion disorders, somatoform disorders, and other neurotic disorders), sleep disorders, eating disorders, and sexual disorders using the International Classification of Diseases (ICD) Tenth Revision codes after the index date. This population-based cohort study was also conducted by the Strengthening the Reporting of Observational Studies in Epidemiology (STROBE) guidelines [16].

### Study population

**Fig. 1:**
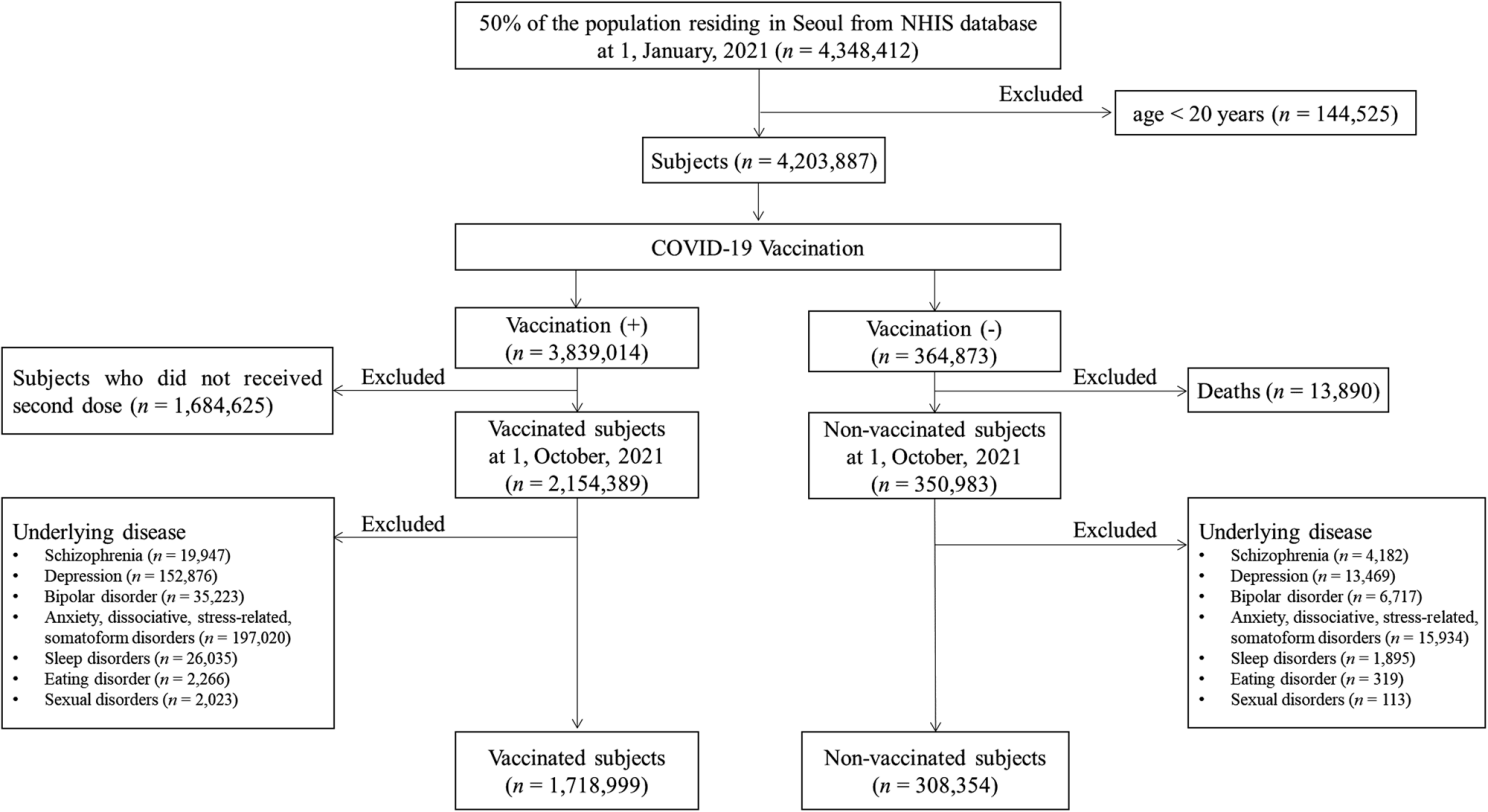
Flowchart of this study.

A total of 4,348,412 individuals living in Seoul, South Korea, constituting 50% of the population, were included and investigated as of January 1, 2021. Individuals under 20 years (n = 144,525) were excluded, leaving 4,203,887 individuals for analysis. We initially divided into two groups based on COVID-19 vaccination and defined as vaccinated group as individuals who received two doses of the COVID-19 vaccine. For the 3,839,014 vaccinated population, we excluded 1,684,625 individuals who did not receive a second dose by 1, October 2021. For 364,873 unvaccinated population, we excluded 13,890 individuals who died on 1, October 2021. The diagnostic records for the year preceding the index date were traced to investigate the causal relationship between vaccine administration and AEs. The occurrence of the target psychiatric disorders was defined as receiving a primary diagnosis of the disease from the day following the index date. Individuals who had received a primary or secondary diagnosis of any target disease for a year prior to index date were excluded from the study. We finally included the participants defined by two groups in this study: the vaccinated group (n = 1,718,999) and the non-vaccinated group (n = 308,354) (Fig. 1).

### Outcome measurements

The primary outcome measure was the cumulative incidence of psychiatric AEs per 10,000 population at one week, two weeks, one month, and three months between two groups. The secondary outcome measures were risks of psychiatric target AEs for COVID-19 vaccination using the odd ratios (ORs) and hazard ratios (HRs). Furthermore, subgroup analyses were also conducted based on gender, age, the number of COVID-19 vaccine doses, the vaccine type (mRNA vaccine, cDNA vaccine, and heterologous vaccination), health insurance level, presence of diabetes mellitus (DM), hypertension (HTN), hyperlipidemia, and chronic obstructive pulmonary disease (COPD). Age, gender, insurance level, Charlson’s comorbidity index (CCI), presence of DM, HTN, hyperlipidemia, and COPD, and prior COVID-19 infection history were extracted using their ICD-19 codes, which were suggested by Sundararajan et al. [17]. The presence of comorbid diseases (i.e., DM, HTN, hyperlipidemia, and COPD), categories of CCI, and the prior COVID-19 infection history were determined based on receiving a primary or secondary diagnosis at least twice within one year before the index date. The National Health Insurance (NHI) premium was used as a proxy for income, as it is proportional to monthly income, encompassing both earnings and capital gains. The income quantiles of the enrolled participants were subdivided into three groups (low-, middle- and high-income groups in medical aid enrollees and the 0–33, 34–66, and 67–100 centiles of NHI enrollees). Detailed information for ICD-10 codes used for analysis is presented in Supplementary Table 1.

### Statistical analysis

Statistical analysis was performed using the SAS Enterprise Guide (version 8.3., SAS Institute, Cary, NC, USA). A normal distribution was confirmed with the Kolmogorov–Smirnov test. Baseline patient characteristics and comorbidities were reported as means ± standard deviation for continuous variables and ratio for categorical variables. Student’s *t* test was performed for continuous variables and the chi-square test for categorical variables. The cumulative incidence was calculated per 10,000 populations. To identify the association between COVID-19 vaccination and psychiatric AEs, a multiple logistic regression model was used for ORs, corresponding to 95% CIs. Cox proportional hazards regression was used to estimate the HRs and 95% CIs. Two-sided *p* values of 0.05 or less were considered to indicate statistical significance.

## Results

### The participants’ characteristics

In total, 2,027,353 subjects were included in this study. Among them, 308,354 (15.21%) had not received the COVID-19 vaccine (i.e., non-vaccinated subjects, no vaccination group in this study), whereas 1,718,999 (84.79%) were vaccinated against COVID-19 (i.e., vaccinated subjects, the vaccinated group in this study). The baseline characteristics of the vaccinated and non-vaccinated groups are presented in Table 1.

**Table 1:** Baseline characteristics of the patients stratified by COVID-19 vaccination in Seoul, South Korea.

	Total (*n* = 2,027,353)	Vaccination		*p*
		No (*n* = 308,354)	Yes (*n* = 1,718,999)	
Gender (*n*)				0.028
Male	955,180 (47.11%)	144,719 (46.93%)	810,461 (47.15%)	
Female	1,072,173 (52.89%)	163,635 (53.07%)	908,538 (52.85%)	
Age (years)	53.07 ± 16.70^a^	44.18 ± 16.28^a^	54.67 ± 16.26^a^	<0.001* <0.001
20–29 years (*n*)	248,526 (12.26%)	59,155 (19.18%)	189,371 (11.02%)	
30–39 years (*n*)	228,104 (11.25%)	82,599 (26.79%)	145,505 (8.46%)	
40–49 years (*n*)	271,478 (13.39%)	67,299 (21.83%)	204,179 (11.88%)	
50–59 years (*n*)	495,117 (24.42%)	43,916 (14.24%)	451,201 (26.25%)	
60–69 years (*n*)	463,848 (22.88%)	30,091 (9.76%)	433,757 (25.23%)	
70–79 years (*n*)	226,411 (11.17%)	13,440 (4.36%)	212,971 (12.39%)	
≥80 years (*n*)	93,869 (4.63%)	11,854 (3.84%)	82,015 (4.77%)	
Insurance level (*n*)				<0.001
Low	508,737 (25.09%)	88,192 (28.60%)	420,545 (24.46%)	
Moderate	571,150 (28.17%)	98,141 (31.83%)	473,009 (27.52%)	
High	947,466 (46.73%)	122,021 (39.57%)	825,445 (48.02%)	
CCI (*n*)				<0.001
0	1,419,603 (70.02%)	267,773 (86.84%)	1,151,830 (67.01%)	
1	325,012 (16.03%)	20,352 (6.60%)	304,660 (17.72%)	
≥2	282,738 (13.95%)	20,229 (6.56%)	262,509 (15.27%)	
Comorbidity (*n*)				
DM	291,780 (14.39%)	15,993 (5.19%)	275,787 (16.04%)	<0.001
Hyperlipidemia	594,939 (29.35%)	30,622 (9.93%)	564,317 (32.83%)	<0.001
HTN	520,673 (25.68%)	26,531 (8.60%)	494,142 (28.75%)	<0.001
COPD	68,185 (3.36%)	4,956 (1.61%)	63,229 (3.68%)	<0.001
Prior COVID-19 infection (*n*)	15,090 (0.74%)	3,099 (1.01%)	11,991 (0.70%)	< 0.001
1st vaccination product (*n*)				
AZD1222	712,187 (41.43%)		712,187 (41.43%)	
BNT162b2	980,129 (57.02%)		980,129 (57.02%)	
mRNA-1273	26,676 (1.55%)		26,676 (1.55%)	
JNJ-78436735	7 (0.00%)		7 (0.00%)	
2nd vaccination product (*n*)				
AZD1222	593,773 (34.54%)		593,773 (34.54%)	
BNT162b2	1,098,521 (63.90%)		1,098,521 (63.90%)	
mRNA-1273	26,698 (1.55%)		26,698 (1.55%)	
JNJ-78436735	7 (0.00%)		7 (0.00%)	
1st–2nd vaccination product (*n*)				
AZD1222–AZD1222	593,766 (34.54%)		593,766 (34.54%)	
AZD1222–BNT162b2	118,413 (6.89%)		118,413 (6.89%)	
AZD1222–mRNA-1273	2 (0.00%)		2 (0.00%)	
AZD1222–JNJ-78436735	6 (0.00%)		6 (0.00%)	
BNT162b2–AZD1222	4 (0.00%)		4 (0.00%)	
BNT162b2–BNT162b2	980,100 (57.02%)		980,100 (57.02%)	
BNT162b2–mRNA-1273	24 (0.00%)		24 (0.00%)	
BNT162b2–JNJ-78436735	1 (0.00%)		1 (0.00%)	
mRNA-1273–BNT162b2	4 (0.00%)		4 (0.00%)	
mRNA-1273–mRNA-1273	26,672 (1.55%)		26,672 (1.55%)	
JNJ-78436735–AZD1222	3 (0.00%)		3 (0.00%)	
JNJ-78436735–BNT162b2	4 (0.00%)		4 (0.00%)	
1st–2nd vaccination type (*n*)				
No vaccination	308,354 (15.21%)	308,354 (100.00%)		
Only mRNA vaccine	1,006,805 (49.66%)		1,006,805 (58.57%)	
Only cDNA vaccine	593,766 (29.29%)		593,766 (34.54%)	
Heterologous vaccination	118,428 (5.84%)		118,428 (6.89%)	
Vaccination interval (days)	50.71 ± 23.16		50.71 ± 23.16	

*n* number, *CCI* Charson’s comorbidity index, *DM* Diabetic mellitus, *HTN* Hypertension, *COPD* Chronic obstructive pulmonary diseases, *AZD*-1222 AstraZeneca ChAdOx1-S recombinant vaccine, *BNT162b2* Pfizer-BioNTech Comirnaty, *mRNA-1273* Moderna Spikevax, *JNJ-78436735* Janssen/Johnson and Johnson COVID-19 Vaccine.

^a^All values expressed as mean ± standard deviation.

### The cumulative incidences per 10,000 of psychiatric AEs following the COVID-19 vaccination

The cumulative incidence of the psychiatric AEs at three months was 0.51 (95% CI, 0.40–0.62) vs 1.98 (95% CI, 1.48–2.47) for schizophrenia, 18.30 (95% CI, 17.66-18.93) vs 14.24 (95% CI, 12.91–15.57) for depression, 0.79 (95% CI, 0.66–0.92) vs 1.39 (95% CI, 0.98–1.81) for bipolar disorder, 28.41 (95% CI, 27.62–29.21) vs 20.27 (95% CI, 18.68–21.86) for anxiety, dissociative, stress-related, and somatoform disorders, 0.30 (95% CI, 0.22–0.38) vs 0.32 (95% CI, 0.12–0.53) for eating disorder, 28.85 (95% CI, 28.05–29.96) vs 12.19 (95% CI, 10.96–13.43) for sleep disorders, 0.27 (95% CI, 0.19–0.34) vs 0.03 (95% CI, 0.00–0.10) for sexual disorders between the vaccinated group and non-vaccinated group. Therefore, the cumulative incidences of schizophrenia (*p* < 0.001) and bipolar disorder (*p* = 0.002) were significantly lower in the vaccinated group than in the non-vaccinated group. Meanwhile, depression (*p* < 0.001), anxiety, dissociative, stress-related, and somatoform disorders (*p* < 0.001), sleep disorders (*p* < 0.001), and sexual disorders (*p* = 0.007) showed significantly higher cumulative incidence in the vaccinated group than in the non-vaccinated group. There was no statistical difference in the cumulative incidence of eating disorders at three months between the two groups (*p* = 0.724). Detailed information of cumulative incidence was presented in Table 2.

**Table 2:** The cumulative incidence of psychiatric adverse events (AEs) following COVID-19 vaccination.

Diseases	Vaccination	Total number	One week				Two weeks				One month				Three months			
			event	I	95% CI	*p*	event	I	95% CI	*p*	event	I	95% CI	*p*	event	I	95% CI	*p*
Schizophrenia	No	308,354	5	0.16	0.02–0.30	0.017	7	0.23	0.06–0.40	0.018	24	0.78	0.47–1.09	<0.001	61	1.98	1.48–2.47	<0.001
	Yes	1,718,999	6	0.03	0.01–0.06		12	0.07	0.03–0.11		26	0.15	0.09–0.21		88	0.51	0.40–0.62	
Depression	No	308,354	26	0.84	0.52–1.17	0.290	54	1.75	1.28–2.22	0.097	142	4.61	3.85–5.36	0.026	439	14.24	12.91–15.57	<0.001
	Yes	1,718,999	184	1.07	0.92–1.23		386	2.25	2.02–2.47		965	5.61	5.26–5.97		3145	18.30	17.66–18.93	
Bipolar disorder	No	308,354	2	0.06	0.00–0.15	0.655	4	0.13	0.00–0.26	0.517	9	0.29	0.10–0.48	0.299	43	1.39	0.98–1.81	0.002
	Yes	1,718,999	8	0.05	0.01–0.08		15	0.09	0.04–0.13		35	0.20	0.14–0.27		136	0.79	0.66–0.92	
Anxiety, dissociative, stress-related, somatoform disorders	No	308,354	48	1.56	1.12–2.00	0.014	95	3.08	2.46–3.70	<0.001	208	6.75	5.83–7.66	<0.001	625	20.27	18.68–21.86	<0.001
	Yes	1,718,999	389	2.26	2.04–2.49		766	4.46	4.14–4.77		1641	9.55	9.08–10.01		4884	28.41	27.62–29.21	
Sleep disorders	No	308,354	29	0.94	0.60–1.28	<0.001	58	1.88	1.40–2.36	<0.001	132	4.28	3.55–5.01	<0.001	376	12.19	10.96–13.43	<0.001
	Yes	1,718,999	330	1.92	1.71–2.13		709	4.12	3.82–4.43		1544	8.98	8.53–9.43		4960	28.85	28.05–29.66	
Eating disorders	No	308,354	2	0.06	0.00–0.15	0.169	2	0.06	0.00–0.15	0.349	3	0.10	0.00–0.21	0.735	10	0.32	0.12–0.53	0.724
	Yes	1,718,999	3	0.02	0.00–0.04		6	0.03	0.01–0.06		14	0.08	0.04–0.12		51	0.30	0.22–0.38	
Sexual disorders	No	308,354	0	0.00	0.00–0.00	1.000	0	0.00	0.00–0.00	0.617	0	0.00	0.00–0.00	0.154	1	0.03	0.00–0.10	0.007
	Yes	1,718,999	1	0.01	0.00–0.02		8	0.05	0.01–0.08		16	0.09	0.05–0.14		46	0.27	0.19–0.34	

*I* cumulative incidence, *CI* confidence interval.

### The cumulative incidences per 10,000 of psychiatric AEs according to vaccine types

**Fig. 2:**
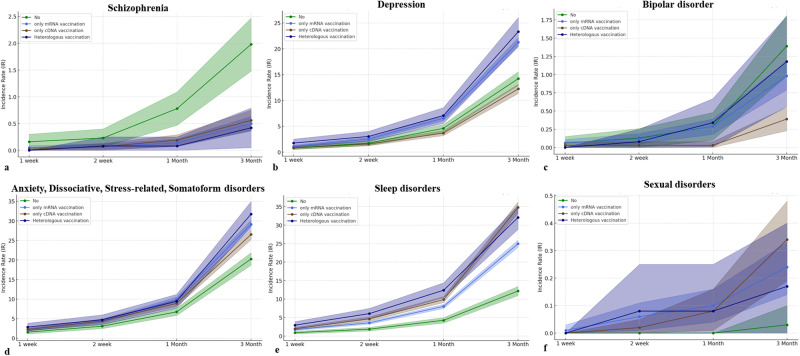
Cumulative incidence rates of mental disorders following COVID-19 vaccination according to vaccine type. **a** Schizophrenia. **b** Depression. **c** Bipolar disorder. **d** Anxiety, dissociative, stress-related, and somatoform disorders. **e** Sleep disorder. **f** Sexual disorders.

Our data was also stratified by vaccine type including vaccination using mRNA-based vaccine only (only mRNA vaccine), vaccination using cDNA-based vaccine only (only cDNA vaccine), and heterologous vaccination, which were compared by non-vaccinated group. For decreased incidences of schizophrenia and bipolar disorder following COVID-19 vaccination, schizophrenia showed the lowest cumulative incidence at three months in the case of heterologous vaccination (0.42; 95% CI, 0.05–0.79) compared to other vaccine types with statistical significances (*p* < 0.001). The lowest cumulative incidence of bipolar disorder at three months was observed in the case of vaccination using only cDNA vaccine (0.39; 95% CI, 0.23-0.55) with a statistical difference (*p* < 0.001). For increased incidences of depression, anxiety, dissociative, stress-related, and somatoform disorders, sleep disorders, and sexual disorders following COVID-19 vaccination, the highest cumulative incidence of depression at three months was significantly observed in heterologous vaccination (23.31; 95% CI, 20.56–26.05) compared to other vaccine types (*p* < 0.001). However, the cumulative incidence of depression at three months was lower in the case of vaccination using only cDNA vaccine (12.26; 95% CI, 11.37–13.15) than in the non-vaccinated group (14.24; 95% CI, 12.91–15.57) with statistical differences (*p* = 0.014). The incidence of anxiety, dissociative, stress-related, and somatoform disorders was significantly observed to be highest in the case of heterologous vaccination (31.75; 95% CI, 28.55–34.95), followed by only mRNA vaccination (29.13; 95% CI, 28.08–30.18) and only cDNA vaccination (26.53; 95% CI, 25.22–27.83) (*p* < 0.001). The incidence of sleep disorders at three months was significantly observed to be high level in both cases of only cDNA vaccination (34.78; 95% CI, 33.28–36.28), and heterologous vaccination (32.09; 95% CI, 28.87–35.31), followed by only mRNA vaccination (24.98; 95% CI, 24.00–25.96) (*p* < 0.001). The cumulative incidences of sexual disorders showed no statistical differences up to one month (*p* > 0.05). At three months, the sexual disorder showed differences in cumulative incidence according to vaccine types (*p* = 0.04). There was no statistical difference in the cumulative incidence of eating disorders at three months (*p* = 0.785) according to vaccine types. The cumulative incidences of psychiatric AEs according to vaccine type are presented in Fig. 2 and Supplementary Table 2.

### The risks of psychiatric AEs following the COVID-19 vaccination

**Fig. 3:**
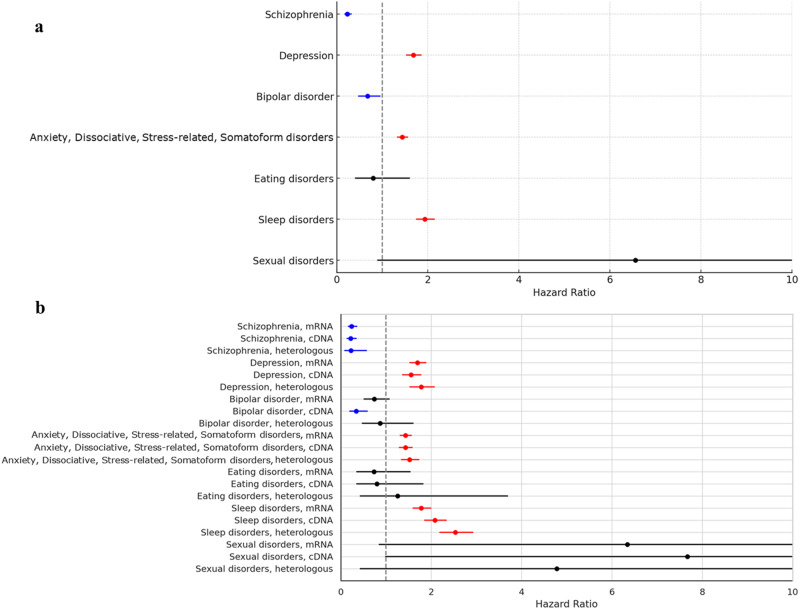
The risk factors for mental disorders in this population. **a** The risks of psychiatric adverse events (AEs) according to COVID-19 vaccination. **b** The risks of psychiatric adverse events according to the COVID-19 vaccine types.

In the Cox proportional hazard model in this study, the HR for COVID-19 vaccination was 0.231 (95% CI, 0.164–0.326) for schizophrenia, 1.683 (95% CI, 1.520–1.863) for depression, 0.672 (95% CI, 0.470–0.962) for bipolar disorder, 1.439 (95% CI, 1.322–1.568) for anxiety, dissociative, stress-related, and somatoform disorders, 0.796 (95% CI, 0.395–1.604) for eating disorders, 1.934 (95% CI, 1.738–2.152) for sleep disorders, and 6.556 (95% CI, 0.890–48.296) for sexual disorders. Therefore, COVID-19 vaccination significantly decreased the risks of occurrence for schizophrenia and bipolar disorder, while significantly increasing the risks for depression, anxiety, dissociative, stress-related, and somatoform disorders, and sleep disorders. There was no effect of COVID-19 vaccination on the occurrence of eating disorders and sexual disorders (Fig. 3a).

In the multivariate logistic model in this study, the ORs of target psychiatric AEs, except for bipolar disorder at one week, two weeks, and one month showed statistical significance, indicating similar patterns for HRs in the Cox proportional hazard model. For bipolar disorder, the OR showed 1.166 (95% CI, 0.240–5.670; *p* = 0.849) at one week, 0.946 (95% CI, 0.304–2.949; *p* = 0.924) at two weeks, 0.982 (95% CI, 0.463–2.079; *p* = 0.962) at one month, and 0.674 (95% CI, 0.471–0.964; *p* = 0.031). The detailed information for ORs of target psychiatric AEs was described in Supplementary Table 3.

### The risks of psychiatric AEs according to the COVID-19 vaccine type

We further assessed the risks of target psychiatric AEs according to the COVID-19 vaccine. For schizophrenia and bipolar disorder which were observed to have lower occurrences following COVID-19 vaccination, the HRs of schizophrenia were 0.239 (95% CI, 0.163–0.352) in only mRNA vaccination, 0.218 (95% CI, 0.138–0.343) in only cDNA vaccination, and 0.227 (95% CI, 0.091–0.566) in heterologous vaccination with statistical significances. On the other hand, the HR of bipolar disorder was statistically significant only in the case of only cDNA vaccination (0.339; 95% CI, 0.196–0.587). For depression, anxiety, dissociative, stress-related, and somatoform disorders, and sleep disorders which were observed to have higher occurrences following COVID-19 vaccination, the HRs of depression (1.777; 95% CI, 1.527–2.067), anxiety, dissociative, stress-related, and somatoform disorders (1.521; 95% CI, 1.338–1.729), and sleep disorders (2.080; 95% CI, 1.855–2.332) showed the highest levels in the case of heterologous vaccination with statistical significances, respectively. There were no statistical differences in risks of eating disorders and sexual disorders according to vaccine types (Fig. 3b).

The ORs of target psychiatric AEs, except for bipolar disorder at one week, two weeks, and one month also showed statistical significance, indicating similar patterns for HRs in the Cox proportional hazard model. For bipolar disorder, the OR for only cDNA vaccination showed 1.581 (95% CI, 0.172–14.496; *p* = 0.685) at one week, 0.459 (95% CI, 0.073–2.893; *p* = 0.407) at two weeks, 0.191 (95% CI, 0.039–0.939; *p* = 0.042) at one month, and 0.340 (95% CI, 0.197–0.589; *p* < 0.001). The detailed information for ORs of target psychiatric AEs according to vaccine types was described in Supplementary Table 3.

### Subgroup analysis for psychiatric AEs based on gender, age, insurance level, and CCI

As age increases, the risks of schizophrenia (HR, 1.027; 95% CI, 1.016–1.039) and sleep disorders (HR, 1.014; 95% CI, 1.012–1.016) significantly increase and the risks of depression (HR, 0.969; 95% CI, 0.966–0.971), bipolar disorder (HR, 0.982; 95% CI, 0.971–0.993), anxiety, dissociative, stress-related, somatoform disorders (HR, 0.988; 95% CI, 0.986–0.990) significantly decrease. Considering the HR values, age does not seem to largely affect the risk of psychiatric AEs. For the insurance level, the HR of schizophrenia at the high insurance level was 0.593 (*p* = 0.007) with statistical differences compared to the low insurance level. The HRs of depression at the high insurance level and middle insurance level were respectively 0.904 (*p* = 0.014) and 0.824 (*p* < 0.001) with statistical differences. There were no statistical differences in other psychiatric disorders for the insurance levels. For CCI, a higher score of CCI more than 2 significantly increased the risks of depression (HR, 1.393; 95% CI, 1.217–1.596), anxiety, dissociative, stress-related, somatoform disorders (HR, 1.440; 95% CI, 1.306–1.587), and sleep disorders (HR, 1.315; 95% CI, 1.198–1.444) than zero score of CCI. Based on these results, women are mostly susceptible to psychiatric AEs (except for sexual disorders) compared to men. Detailed information for Cox proportional hazard model for psychiatric AEs was presented in Supplementary Table 4.

## Discussion

The post-sequelae of COVID-19 and vaccine-related AEs have globally been concerns for mental illness, ranging from mild signs of mental symptoms to psychiatric disorders [18]. During the prolonged COVID-19 pandemic, there has been an emerging trend in complications of COVID-19 infection and vaccination, intensifying the psychosocial burdens [5, 18, 19]. Despite the considerable clinical benefits of the COVID-19 vaccination, it paradoxically made managing psychiatric disorders more challenging due to the contradictory outcomes associated with COVID-19 vaccination [11, 13–15, 18–21]. Here, we conducted a population-based retrospective cohort study for psychiatric AEs after COVID-19 vaccination in Seoul, South Korea. From our cohort between 1,718,999 vaccinated subjects and 308,354 non-vaccinated subjects, we found that the vaccinated subjects showed a significantly higher incidence of depression, anxiety, dissociative, stress-related, and somatoform disorders, sleep disorders, and sexual disorders and a significantly lower incidence of schizophrenia and bipolar disorder than the non-vaccinated subjects. Furthermore, COVID-19 vaccination increased risks of depression, anxiety, dissociative, stress-related, and somatoform disorders and sleep disorders but reduced risks of schizophrenia and bipolar disorder.

Some evidence between COVID-19 and mental illness has gradually grown since the most common symptoms of the long-COVID-19 pandemic were depression/anxiety, psychotic disorder, and cognitive impairment (called brain fog) experienced by 22% of patients within 6 months after COVID-19 infections [4, 14]. However, there have been contradictory reports between mental illness and COVID-19 vaccinations. Chaudhuri et al. reported that vaccination significantly alleviated psychological distress measured by the General Health Questionnaire in the UK Household Longitudinal cohort study [18]. Meanwhile, Balasubramanian et al. reviewed the reports of psychiatric AEs to COVID-19 vaccines, which illustrated 14 cases of psychiatric reactions including psychosis, depression, and anxiety, dissociative, stress-related, and somatoform disorders [15]. To our knowledge, there are scarce studies on the association between vaccines and psychiatric AEs. Therefore, our population-based cohort study provides robust evidence for the psychiatric AEs after COVID-19 vaccinations. Furthermore, our study provided risks of psychiatric AEs according to vaccine type, revealing that the psychiatric disorders (depression, anxiety, dissociative, stress-related, and somatoform disorders, and sleep disorders) with increased risks due to COVID-19 vaccination showed the highest risk in the case of heterogeneous vaccination. Meanwhile, occurrences of schizophrenia consistently decreased risks according to vaccine type but bipolar disorder showed significantly decreased risks from only cDNA vaccination.

Two representative mood disorders, depression, and bipolar disorders, showed contrasting trends for COVID-19 vaccination. The serotonin theory is that depression is caused by an alternation of the hypothalamic-pituitary-adrenal (HPA) axis, particularly serotonin (5-hydroxytryptamine or 5-HT) [22–24]. Along with this theory, the selective serotonin reuptake inhibitor is currently the main drug to treat depression [22]. Serotonin, known as a neurotransmitter, is important to immune systems as the regulator of immune responses and inflammatory processes by central and peripheral mechanisms [22, 24]. For reports of COVID-19 vaccines, the association between adrenal crisis and COVID-19 vaccination has been suggested with the possible risk of heterologous vaccination [25, 26] Therefore, COVID-19 may alternate the HPA axis, which can potentially increase the risk of depression from our study. In our study, HR for bipolar disorder was found to be 0.672 (95% CI, 0.470–0.962) in association with COVID-19 vaccination. However, this result was primarily caused by only cDNA vaccination with a notable lower HR of 0.339 (95% CI, 0.196–0.587). Interestingly, other types of COVID-19 vaccinations did not demonstrate a significant impact on the occurrences of bipolar disorder. These distinctive findings suggested that the differential effects along with vaccine types may be underestimated in mental illness, particularly bipolar disorder [12, 27].

The immune response mediated by COVID-19 vaccination manifests in a variety of ways across different sites in our body [28]. Trougakos et al. described that the AEs following COVID-19 vaccination may related to the proinflammatory action of the lipid nanoparticles or the delivered mRNA and proinflammatory effects of the produced antigens-spike protein and/or its peptides fragments [29]. The COVID-19 vaccination activated proinflammatory cytokines such as interleukin (IL)-1, IL-6, and tumor necrosis factor-α mediated by CD4^+^ T cells [28]. Many immune-related factors have been suggested for the expression of psychosis [13]. The hyperinflammatory status can increase dopamine with N-inhibition of methyl-d-aspartate receptor (NMDAR), which leads to psychosis [30]. Furthermore, an autoimmune response caused by the spike protein and encoded viral protein in the vaccine can be a potential factor in the manifestation of schizophrenia [31, 32]. Of many vaccination methods, Lee et al. described that the heterologous vaccination enhanced B cells and CD4^+^ T cell responses [33]. Proinflammatory effects can be exacerbated by preexisting inflammatory conditions after administration of mRNA lipid nanoparticles [29]. This heightened immune response may influence the occurrence of schizophrenia, as presented in our study. However, the profound pathophysiological mechanisms should be performed in future translational research [27].

Anxiety and stress-related disorders are the main concerns for COVID-19 infection and vaccination, which were widely studied from fear to vaccine hesitancy [34]. Conversion disorders, characterized by paralysis, sensory disturbances, and seizures are associated with alternation of brain function networks [35]. Our study showed increased risks of anxiety, dissociative, stress-related, and somatoform disorders, and sleep disorders, which were heightened by heterologous vaccination. Most patients who were infected with COVID-19 experienced chronic fatigue with mild cognitive impairment (i.e., brain fog) [21, 35, 36]. Abel et al. described that COVID-19 infection increased the risks of fatigue and sleep disorders from the UK primary care data [21]. The mechanism is thought to be caused by a decrease in cerebral blood flow, and it is similar to neuropsychiatric disorders affected by inflammatory processes and immune responses [37]. Since COVID-19 vaccination is also associated with immune responses, both clinicians and vaccinated subjects should be cautious of these manifestations, especially anxiety, dissociative, stress-related, and somatoform disorders, and sleep disorders that can also be affected by COVID-19 vaccination in our study. Therefore, the COVID-19 vaccination increases the manifestation of neurosis-related disorders but decreases that of psychosis-related disorders.

With the increasing evidence of extrapulmonary manifestations including neurological and psychiatric symptoms, COVID-19 infection as well as vaccination may affect the central and peripheral nervous system with profound cellular and molecular mechanisms [6, 38, 39]. The spike protein, especially brain-infiltrating SARS-CoV-2 spike protein has been suggested as an important target for the development of neurological and psychiatric disorders. Several mechanisms involved in spike protein have been proposed at the mice level such as TLR2-mediated depression, TLR4-mediated cognitive dysfunction, and anxiety mediated by non-cell autonomous hippocampal neuronal cell deaths by inducing IL-1β from glial cells [40]. Importantly, a recent study suggested that mRNA vaccines have slippery sequences inducing +1 ribosomal frameshifting products after vaccinations as a consequence of N^1^-methylpseudouridine-induced ribosome stalling [41]. Although the pathogenesis remains unclear, our study suggests that neuroinflammation caused by spike proteins may contribute to occurrences of some psychiatric AEs such as depression and anxiety, dissociative, stress-related, and somatoform disorders. Therefore, future translational research may provide the pathophysiological differences between psychosis and neurosis from the COVID-19 vaccination.

To supplement our hypothesis regarding the mechanism of action after COVID-19 vaccination, we tried further study using the gene sets enrichment analysis (Supplementary Table 5). We found that schizophrenia-related genes share the enrichment pathway for bile acid metabolism. Bile acids prevent the binding of spike protein with angiotensin-converting enzyme II (ACE2) and modulate the expression of ACE2, suggesting the protective role [42]. Our study also showed that COVID-19 vaccination reduced risks of schizophrenia. For depression, as an increased risk after COVID-19 vaccination, the results were shown for the deep interaction of spike protein-related factors such as NLRP3 inflammasome [40]. This supports that the presence of the spike protein plays a crucial role in the manifestation of diseases after COVID-19 vaccination. Regarding the neurosis, the Rap1 signaling was observed as an enrichment pathway in the genes of neuroticisms. It regulates MAPK pathways that are important for SARS-CoV-2 virus replication [43, 44]. However, these findings will have to be validated by future experimental studies.

This study has several limitations. First, we collected the claims data based on ICD-19 codes, which led to potential errors regarding mismatching or misclassification could have occurred. Second, previous studies have suggested the impact of poverty rates on mental disorders but there are differences in the baseline characteristics between the two groups in this study [45, 46]. Although propensity score matching could be a statistical option to overcome this limitation, the real-world data is scarce on the psychiatric AEs following the COVID-19 vaccination so future studies through the process of propensity score matching should be addressed for COVID-19 vaccine-related psychiatric AEs. Meanwhile, both schizophrenia and bipolar disorders often manifest at a young age (under 20 years old) [47]. However, our study protocol didn’t include the adolescent population group because our study protocol has been approved by our IRB to analyze adults more than 20 years old. Furthermore, the COVID-19 vaccination for adolescents in South Korea has been authorized since October 2021, which made it impossible to collect COVID-19 vaccine-related AEs of the population under 20 years old in our study protocol. Thus, future studies for adolescents considering the manifestation of psychiatric disorders will be needed on COVID-19 vaccine-related AEs. Third, we measured psychiatric AEs up to three months following COVID-19 vaccination. This study did not contain the long-term follow-up COVID-19 vaccine-associated AEs. Last, despite being a population-based study targeting the population in Seoul, South Korea, it cannot be generalized to the entire population, globally since psychiatric disorders could be related to ethnic and genetic backgrounds. Therefore, a global-scale real-world study for adverse events of COVID-19 vaccination will be important in the future.

In conclusion, this population-based cohort study revealed that COVID-19 vaccination differentially affects occurrences of psychiatric disorders. It increased the risks of depression, anxiety, dissociative, stress-related, and somatoform disorders, and sleep disorders while reducing the incidence and risk of schizophrenia and bipolar disorder. Our findings suggested that the relationship between COVID-19 vaccination and mental illness may be underestimated along with the complexity of its impact on mental health. Thus, close observation and special caution are necessary for administering additional COVID-19 vaccinations to populations vulnerable to psychiatric AEs.

## Supplementary information

Supplementary tables

## Data Availability

The datasets analyzed during the current study are available through an application to the National Health Insurance Service, South Korea. This protects the confidentiality of the data and ensures that Information Governance is robust.
