# Supplementary material for: Psychiatric adverse events following COVID-19 vaccination: a population-based cohort study in Seoul, South Korea
Source: Mol Psychiatry. 2024 Jun 4;29(11):3635–43. doi: 10.1038/s41380-024-02627-0 (PMC11541197; doi:10.1038/s41380-024-02627-0)
Supplement: Supplementary file 1 — Supplementary tables [file 41380_2024_2627_MOESM1_ESM.docx]

**Supplementary Table 1.** Overview of International Classification of Diseases (ICD)-10 codes in this study

| **Overview of ICD-10 codes used to define psychiatric disorders** | |
| --- | --- |
| **Category** | **ICD-10 codes** |
| Schizophrenia | F20-F29 |
| Depression | F32, F33 |
| Bipolar disorder | F31 |
| Anxiety, Dissociative, Stress-related, Somatoform disorders | F40-F48 |
| Sleep disorders | F51, G47 |
| Eating disorders | F50 |
| Sexual disorders | F52 |
| **Overview of ICD-10 codes used to define comorbidity** | |
| CCI | B20-B24, C0-C3, C40, C41, C43, C45-C49, C5, C6, C70- C85, C91-C93, C95-C97, C883, C887, C889, C900, C901, C940-C943, C947, E10, E11, E13, E14, E102-E104, E112-E114, E132-E134, E142-E144, F00-F02, F051, G81, G46, G450-G452, G454, G458, G459, G820-G822, I21, I22, I50, I60-I66, I69, I71, I252, I670-I679, I681, I682, I688, I739, I790, J40-J47, J60-67, K25-K28, K73, K702, K703, K717, K721, K729, K740, K742-K746, K766, K767, M05, M060, M063, M069, M32, M34, M332, M353, N01, N03, N052-N056, N072-N074, N18, N19, N25, R02, Z958, Z959 |
| DM | E10-E14 |
| HTN | I10-I15 |
| Hyperlipidemia | E78 |
| COPD | J40-J44 |
| COVID-19 infection | U071 |

CCI, Charlson comorbidity index; DM, diabetes mellitus; HTN, hypertension, COPD, chronic obstructive pulmonary disease, COVID-19, coronavirus disease-2019.

**Supplementary Table 2.** The cumulative incidence of psychiatric adverse events (AEs) according to COVID-19 vaccine types

| **Diseases** | **Vaccine types** | **Total**  **number** | **One week** | | | | **Two weeks** | | | | **One month** | | | | | **Three months** | | | | |
| --- | --- | --- | --- | --- | --- | --- | --- | --- | --- | --- | --- | --- | --- | --- | --- | --- | --- | --- | --- | --- |
|  |  |  | **event** | **I** | **95% CI** | ***p*** | **event** | **I** | **95% CI** | ***p*** | **event** | **I** | **95% CI** | ***p*** | **event** | | **I** | **95% CI** | ***p*** |  |
| Schizophrenia | No | 308,354 | 5 | 0.16 | 0.02-0.30 | 0.032 | 7 | 0.23 | 0.06-0.40 | 0.074 | 24 | 0.78 | 0.47-1.09 | < 0.001 | 61 | | 1.98 | 1.48-2.47 | < 0.001 |  |
|  | Only mRNA vaccine | 1,006,805 | 5 | 0.05 | 0.01-0.09 |  | 7 | 0.07 | 0.02-0.12 |  | 14 | 0.14 | 0.07-0.21 |  | 50 | | 0.50 | 0.36-0.63 |  |  |
|  | Only cDNA vaccine | 593,766 | 1 | 0.02 | 0.00-0.05 |  | 4 | 0.07 | 0.00-0.13 |  | 11 | 0.19 | 0.08-0.29 |  | 33 | | 0.56 | 0.37-0.75 |  |  |
|  | Heterologous | 118,428 | 0 | 0.00 | 0.00-0.00 |  | 1 | 0.08 | 0.00-0.25 |  | 1 | 0.08 | 0.00-0.25 |  | 5 | | 0.42 | 0.05-0.79 |  |  |
| Depression | No | 308,354 | 26 | 0.84 | 0.52-1.17 | 0.010 | 54 | 1.75 | 1.28-2.22 | < 0.001 | 142 | 4.61 | 3.85-5.36 | < 0.001 | 439 | | 14.24 | 12.91-15.57 | < 0.001 |  |
|  | Only mRNA vaccine | 1,006,805 | 115 | 1.14 | 0.93-1.35 |  | 256 | 2.54 | 2.23-2.85 |  | 662 | 6.58 | 6.07-7.08 |  | 2,141 | | 21.27 | 20.37-22.17 |  |  |
|  | Only cDNA vaccine | 593,766 | 48 | 0.81 | 0.58-1.04 |  | 94 | 1.58 | 1.26-1.90 |  | 219 | 3.69 | 3.20-4.18 |  | 728 | | 12.26 | 11.37-13.15 |  |  |
|  | Heterologous | 118,428 | 21 | 1.77 | 1.01-2.53 |  | 36 | 3.04 | 2.05-4.03 |  | 84 | 7.09 | 5.58-8.61 |  | 276 | | 23.31 | 20.56-26.05 |  |  |
| Bipolar disorder | No | 308,354 | 2 | 0.06 | 0.00-0.15 | 0.742 | 4 | 0.13 | 0.00-0.26 | 0.332 | 9 | 0.29 | 0.10-0.48 | 0.004 | 43 | | 1.39 | 0.98-1.81 | < 0.001 |  |
|  | Only mRNA vaccine | 1,006,805 | 6 | 0.06 | 0.01-0.11 |  | 12 | 0.12 | 0.05-0.19 |  | 29 | 0.29 | 0.18-0.39 |  | 99 | | 0.98 | 0.79-1.18 |  |  |
|  | Only cDNA vaccine | 593,766 | 2 | 0.03 | 0.00-0.08 |  | 2 | 0.03 | 0.00-0.08 |  | 2 | 0.03 | 0.00-0.08 |  | 23 | | 0.39 | 0.23-0.55 |  |  |
|  | Heterologous | 118,428 | 0 | 0.00 | 0.00-0.00 |  | 1 | 0.08 | 0.00-0.25 |  | 4 | 0.34 | 0.01-0.67 |  | 14 | | 1.18 | 0.56-1.80 |  |  |
| Anxiety, dissociative, stress-related, somatoform disorders | No | 308,354 | 48 | 1.56 | 1.12-2.00 | 0.040 | 95 | 3.08 | 2.46-3.70 | 0.008 | 208 | 6.75 | 5.83-7.66 | < 0.001 | 625 | | 20.27 | 18.68-21.86 | < 0.001 |  |
|  | Only mRNA vaccine | 1,006,805 | 221 | 2.20 | 1.91-2.48 |  | 449 | 4.46 | 4.05-4.87 |  | 1,000 | 9.93 | 9.32-10.55 |  | 2,933 | | 29.13 | 28.08-30.18 |  |  |
|  | Only cDNA vaccine | 593,766 | 134 | 2.26 | 1.87-2.64 |  | 261 | 4.40 | 3.86-4.93 |  | 529 | 8.91 | 8.15-9.67 |  | 1,575 | | 26.53 | 25.22-27.83 |  |  |
|  | Heterologous | 118,428 | 34 | 2.87 | 1.91-3.84 |  | 56 | 4.73 | 3.49-5.97 |  | 112 | 9.46 | 7.71-11.21 |  | 376 | | 31.75 | 28.55-34.95 |  |  |
| Sleep disorders | No | 308,354 | 29 | 0.94 | 0.60-1.28 | < 0.001 | 58 | 1.88 | 1.40-2.36 | < 0.001 | 132 | 4.28 | 3.55-5.01 | < 0.001 | 376 | | 12.19 | 10.96-13.43 | < 0.001 |  |
|  | Only mRNA vaccine | 1,006,805 | 176 | 1.75 | 1.49-2.01 |  | 361 | 3.59 | 3.22-3.96 |  | 810 | 8.05 | 7.49-8.60 |  | 2,515 | | 24.98 | 24.00-25.96 |  |  |
|  | Only cDNA vaccine | 593,766 | 119 | 2.00 | 1.64-2.36 |  | 276 | 4.65 | 4.10-5.20 |  | 587 | 9.89 | 9.09-10.69 |  | 2,065 | | 34.78 | 33.28-36.28 |  |  |
|  | Heterologous | 118,428 | 35 | 2.96 | 1.98-3.93 |  | 72 | 6.08 | 4.68-7.48 |  | 147 | 12.41 | 10.41-14.42 |  | 380 | | 32.09 | 28.87-35.31 |  |  |
| Eating disorders | No | 308,354 | 2 | 0.06 | 0.00-0.15 | 0.466 | 2 | 0.06 | 0.00-0.15 | 0.707 | 3 | 0.10 | 0.00-0.21 | 0.721 | 10 | | 0.32 | 0.12-0.53 | 0.785 |  |
|  | Only mRNA vaccine | 1,006,805 | 2 | 0.02 | 0.00-0.05 |  | 3 | 0.03 | 0.00-0.06 |  | 7 | 0.07 | 0.02-0.12 |  | 27 | | 0.27 | 0.17-0.37 |  |  |
|  | Only cDNA vaccine | 593,766 | 1 | 0.02 | 0.00-0.05 |  | 3 | 0.05 | 0.00-0.11 |  | 5 | 0.08 | 0.01-0.16 |  | 19 | | 0.32 | 0.18-0.46 |  |  |
|  | Heterologous | 118,428 | 0 | 0.00 | 0.00-0.00 |  | 0 | 0.00 | 0.00-0.00 |  | 2 | 0.17 | 0.00-0.40 |  | 5 | | 0.42 | 0.05-0.79 |  |  |
| Sexual disorders | No | 308,354 | 0 | 0.00 | 0.00-0.00 | 0.798 | 0 | 0.00 | 0.00-0.00 | 0.305 | 0 | 0.00 | 0.00-0.00 | 0.393 | 1 | | 0.03 | 0.00-0.10 | 0.040 |  |
|  | Only mRNA vaccine | 1,006,805 | 1 | 0.01 | 0.00-0.03 |  | 6 | 0.06 | 0.01-0.11 |  | 10 | 0.10 | 0.04-0.16 |  | 24 | | 0.24 | 0.14-0.33 |  |  |
|  | Only cDNA vaccine | 593,766 | 0 | 0.00 | 0.00-0.00 |  | 1 | 0.02 | 0.00-0.05 |  | 5 | 0.08 | 0.01-0.16 |  | 20 | | 0.34 | 0.19-0.48 |  |  |
|  | Heterologous | 118,428 | 0 | 0.00 | 0.00-0.00 |  | 1 | 0.08 | 0.00-0.25 |  | 1 | 0.08 | 0.00-0.25 |  | 2 | | 0.17 | 0.00-0.40 |  |  |

I, cumulative incidence; CI, confidence interval.

**Supplementary Table 3.** Multivariate logistic model between target psychiatric adverse events (AEs) and COVID-19 vaccination

| **Diseases** | **Variables** | **Values** | **One week** | | | **Two weeks** | | | **One month** | | | **Three months** | | |
| --- | --- | --- | --- | --- | --- | --- | --- | --- | --- | --- | --- | --- | --- | --- |
|  |  |  | **OR** | **95% CI** | ***p*** | **OR** | **95% CI** | ***p*** | **OR** | **95% CI** | ***p*** | **OR** | **95% CI** | ***p*** |
| Schizophrenia | COVID-19 vaccination | Yes | 0.196 | 0.055-0.704 | 0.012 | 0.265 | 0.098-0.716 | 0.009 | 0.174 | 0.097-0.312 | < 0.001 | 0.232 | 0.164-0.327 | < 0.001 |
|  | Control | No |  | | | | | | | | | | | |
|  | Vaccine type | Only mRNA vaccination | 0.274 | 0.076-0.988 | 0.048 | 0.272 | 0.092-0.803 | 0.018 | 0.168 | 0.086-0.331 | < 0.001 | 0.240 | 0.164-0.353 | < 0.001 |
|  |  | Only cDNA vaccination | 0.083 | 0.009-0.809 | 0.032 | 0.226 | 0.059-0.862 | 0.029 | 0.196 | 0.091-0.422 | < 0.001 | 0.219 | 0.139-0.344 | < 0.001 |
|  |  | Heterologous vaccination |  |  |  | 0.369 | 0.045-3.016 | 0.353 | 0.113 | 0.015-0.834 | 0.033 | 0.228 | 0.091-0.568 | 0.002 |
| Depression | COVID-19 vaccination | Yes | 2.872 | 0.688-11.994 | 0.148 | 3.755 | 1.178-11.972 | 0.025 | 2.715 | 1.380-5.342 | 0.004 | 2.729 | 1.786-4.168 | < 0.001 |
|  | Control | No |  |  |  |  |  |  |  |  |  |  |  |  |
|  | Vaccine type | Only mRNA vaccination | 1.563 | 1.018-2.400 | 0.041 | 1.660 | 1.235-2.231 | 0.001 | 1.606 | 1.337-1.928 | < 0.001 | 1.699 | 1.532-1.885 | < 0.001 |
|  |  | Only cDNA vaccination | 1.797 | 1.065-3.032 | 0.028 | 1.769 | 1.227-2.551 | 0.002 | 1.447 | 1.149-1.822 | 0.002 | 1.558 | 1.369-1.773 | < 0.001 |
|  |  | Heterologous vaccination | 2.278 | 1.277-4.062 | 0.005 | 1.897 | 1.241-2.900 | 0.003 | 1.655 | 1.261-2.170 | < 0.001 | 1.779 | 1.529-2.070 | < 0.001 |
| Bipolar disorder | COVID-19 vaccination | Yes | 1.166 | 0.240-5.670 | 0.849 | 0.946 | 0.304-2.949 | 0.924 | 0.982 | 0.463-2.079 | 0.962 | 0.674 | 0.471-0.964 | 0.031 |
|  | Control | No |  |  |  |  |  |  |  |  |  |  |  |  |
|  | Vaccine type | Only mRNA vaccination | 1.224 | 0.245-6.124 | 0.806 | 1.066 | 0.338-3.358 | 0.914 | 1.129 | 0.530-2.408 | 0.753 | 0.745 | 0.517-1.073 | 0.114 |
|  |  | Only cDNA vaccination | 1.581 | 0.172-14.496 | 0.685 | 0.459 | 0.073-2.893 | 0.407 | 0.191 | 0.039-0.939 | 0.042 | 0.340 | 0.197-0.589 | < 0.001 |
|  |  | Heterologous vaccination | 0.000 | 0.000-.00 | 0.978 | 0.757 | 0.084-6.839 | 0.804 | 1.271 | 0.389-4.146 | 0.691 | 0.873 | 0.476-1.600 | 0.660 |
| Anxiety, dissociative, stress-related, somatoform disorders | COVID-19 vaccination | Yes | 1.509 | 1.109-2.052 | 0.009 | 1.477 | 1.187-1.838 | < 0.001 | 1.430 | 1.233-1.659 | < 0.001 | 1.444 | 1.325-1.573 | < 0.001 |
|  | Control | No |  |  |  |  |  |  |  |  |  |  |  |  |
|  | Vaccine type | Only mRNA vaccination | 1.429 | 1.042-1.960 | 0.027 | 1.449 | 1.158-1.813 | 0.001 | 1.451 | 1.247-1.688 | < 0.001 | 1.436 | 1.315-1.567 | < 0.001 |
|  |  | Only cDNA vaccination | 1.653 | 1.155-2.364 | 0.006 | 1.564 | 1.213-2.016 | 0.001 | 1.397 | 1.175-1.661 | < 0.001 | 1.434 | 1.297-1.585 | < 0.001 |
|  |  | Heterologous vaccination | 1.805 | 1.162-2.805 | 0.009 | 1.494 | 1.073-2.081 | 0.017 | 1.352 | 1.074-1.702 | 0.010 | 1.525 | 1.342-1.734 | < 0.001 |
| Sleep disorders | COVID-19 vaccination | Yes | 1.782 | 1.210-2.626 | 0.003 | 1.868 | 1.422-2.453 | < 0.001 | 1.809 | 1.509-2.169 | < 0.001 | 1.942 | 1.745-2.160 | < 0.001 |
|  | Control | No |  |  |  |  |  |  |  |  |  |  |  |  |
|  | Vaccine type | Only mRNA vaccination | 1.677 | 1.126-2.496 | 0.011 | 1.697 | 1.282-2.246 | < 0.001 | 1.684 | 1.398-2.028 | < 0.001 | 1.789 | 1.603-1.996 | < 0.001 |
|  |  | Only cDNA vaccination | 1.625 | 1.063-2.485 | 0.025 | 1.853 | 1.380-2.490 | < 0.001 | 1.778 | 1.460-2.166 | < 0.001 | 2.089 | 1.863-2.342 | < 0.001 |
|  |  | Heterologous vaccination | 3.066 | 1.872-5.024 | < 0.001 | 3.159 | 2.233-4.468 | < 0.001 | 2.826 | 2.232-3.577 | < 0.001 | 2.541 | 2.202-2.932 | < 0.001 |
| Eating disorders | COVID-19 vaccination | Yes | 0.288 | 0.042-1.989 | 0.207 | 0.458 | 0.083-2.521 | 0.369 | 0.742 | 0.202-2.719 | 0.652 | 0.801 | 0.397-1.612 | 0.534 |
|  | Control | No |  |  |  |  |  |  |  |  |  |  |  |  |
|  | Vaccine type | Only mRNA vaccination | 0.308 | 0.041-2.318 | 0.253 | 0.427 | 0.068-2.696 | 0.366 | 0.637 | 0.160-2.536 | 0.522 | 0.737 | 0.352-1.542 | 0.417 |
|  |  | Only cDNA vaccination | 0.344 | 0.020-5.849 | 0.460 | 0.699 | 0.089-5.486 | 0.733 | 0.716 | 0.150-3.415 | 0.675 | 0.806 | 0.355-1.828 | 0.605 |
|  |  | Heterologous vaccination |  |  |  |  |  |  | 1.605 | 0.266-9.672 | 0.606 | 1.264 | 0.431-3.706 | 0.670 |
| Sexual disorders | COVID-19 vaccination | Yes |  |  |  |  |  |  |  |  |  | 6.592 | 0.895-48.544 | 0.064 |
|  | Control | No |  |  |  |  |  |  |  |  |  |  |  |  |
|  | Vaccine type | Only mRNA vaccination |  |  |  |  |  |  |  |  |  | 6.370 | 0.852-47.612 | 0.071 |
|  |  | Only cDNA vaccination |  |  |  |  |  |  |  |  |  | 7.710 | 0.998-59.551 | 0.050 |
|  |  | Heterologous vaccination |  |  |  |  |  |  |  |  |  | 4.801 | 0.433-53.276 | 0.201 |

OR, odd ratio; CI, confidence interval.

**Supplementary Table 4.** Cox proportional hazard model for target psychiatric adverse events (AEs) based on gender, age, insurance level, and Charlson comorbidity index.

| **Diseases** | **Variables** | **Values** | **HR** | **95% CI** | ***p*** |  | **Diseases** | **Variables** | **Values** | **HR** | **95% CI** | ***p*** |
| --- | --- | --- | --- | --- | --- | --- | --- | --- | --- | --- | --- | --- |
| Schizophrenia | Gender | Female | 1.411 | 1.013-1.967 | 0.042 |  | Sleep disorders | Gender | Female | 1.131 | 1.071-1.194 | < 0.001 |
|  | Age |  | 1.027 | 1.016-1.039 | < 0.001 |  |  | Age |  | 1.014 | 1.012-1.016 | < 0.001 |
|  | Insurance level | Low |  | | |  |  | Insurance level | Low |  |  |  |
|  |  | Middle | 0.783 | 0.523-1.173 | 0.236 |  |  |  | Middle | 0.931 | 0.863-1.005 | 0.065 |
|  |  | High | 0.593 | 0.405-0.869 | 0.007 |  |  |  | High | 1.043 | 0.977-1.113 | 0.210 |
|  | CCI | 0 |  | | |  |  | CCI | 0 |  | | |
|  |  | 1 | 0.832 | 0.458-1.512 | 0.546 |  |  |  | 1 | 1.168 | 1.076-1.268 | < 0.001 |
|  |  | 2 ≤ | 0.824 | 0.416-1.631 | 0.579 |  |  |  | 2 ≤ | 1.315 | 1.198-1.444 | < 0.001 |
| Depression | Gender | Female | 1.865 | 1.737-2.002 | < 0.001 |  | Eating disorders | Gender | Female | 2.877 | 1.601-5.172 | < 0.001 |
|  | Age |  | 0.969 | 0.966-0.971 | < 0.001 |  |  | Age |  | 1.006 | 0.987-1.025 | 0.568 |
|  | Insurance level | Low |  | | |  |  | Insurance level | Low |  | | |
|  |  | Middle | 0.824 | 0.754-0.901 | < 0.001 |  |  |  | Middle | 1.032 | 0.508-2.096 | 0.930 |
|  |  | High | 0.904 | 0.835-0.979 | 0.014 |  |  |  | High | 1.171 | 0.629-2.180 | 0.619 |
|  | CCI | 0 |  | | |  |  | CCI | 0 |  | | |
|  |  | 1 | 1.243 | 1.112-1.391 | < 0.001 |  |  |  | 1 | 0.785 | 0.313-1.967 | 0.606 |
|  |  | 2 ≤ | 1.393 | 1.217-1.596 | < 0.001 |  |  |  | 2 ≤ | 2.316 | 0.997-5.380 | 0.051 |
| Bipolar disorder | Gender | Female | 1.400 | 1.033-1.897 | 0.030 |  | Sexual disorders | Gender | Female | 0.020 | 0.003-0.144 | < 0.001 |
|  | Age |  | 0.982 | 0.971-0.993 | 0.001 |  |  | Age |  | 1.009 | 0.987-1.032 | 0.427 |
|  | Insurance level | Low |  | | |  |  | Insurance level | Low |  | | |
|  |  | Middle | 0.835 | 0.573-1.218 | 0.350 |  |  |  | Middle | 0.737 | 0.290-1.870 | 0.520 |
|  |  | High | 0.711 | 0.499-1.013 | 0.059 |  |  |  | High | 1.380 | 0.670-2.846 | 0.383 |
|  | CCI | 0 |  | | |  |  | CCI | 0 |  | | |
|  |  | 1 | 1.145 | 0.683-1.921 | 0.607 |  |  |  | 1 | 0.934 | 0.369-2.363 | 0.885 |
|  |  | 2 ≤ | 1.702 | 0.954-3.035 | 0.072 |  |  |  | 2 ≤ | 1.189 | 0.414-3.418 | 0.748 |
| Anxiety, dissociative, stress-related, somatoform disorders | Gender | Female | 1.488 | 1.408-1.572 | < 0.001 |  | HR, hazard ratio; CI, confidence interval; CCI, Charlson comorbidity index. | | | | | |
|  | Age |  | 0.988 | 0.986-0.990 | < 0.001 |  |  |  |  |  |  |  |
|  | Insurance level | Low |  | | |  |  |  |  |  |  |  |
|  |  | Middle | 0.959 | 0.890-1.032 | 0.261 |  |  |  |  |  |  |  |
|  |  | High | 1.058 | 0.991-1.129 | 0.094 |  |  |  |  |  |  |  |
|  | CCI | 0 |  | | |  |  |  |  |  |  |  |
|  |  | 1 | 1.299 | 1.195-1.412 | < 0.001 |  |  |  |  |  |  |  |
|  |  | 2 ≤ | 1.440 | 1.306-1.587 | < 0.001 |  |  |  |  |  |  |  |

**Supplementary Table 5.** Gene sets and functional analysis for this study

| **Methods** | The gene sets and functional analysis were performed with Enrichr (https://maayanlab.cloud/Enrichr/) [1]. With the psychiatric disorders that are mainly related with our study, the target gene sets for this enrichment analysis were investigated for schizophrenia, depression, and neuroticism. Schizophrenia: GLT8D1, PBRM1, CYP7B1, FAM114A2, GATAD2A, FMBT1 , C12orf65, TRANK1, ACTR1B [2], Depression: NEGR1, EP300, NICN1, MLF1, ZKSCAN8, ITPR3, RP11-220I1.5, NLGN1, FURIN, TRAF3, CSE1L [3], Neuroticism: GRIK3, ENAH, SBF2, DRD2, GPC6, LINGO1, PAFAH1B1, CELF4, L3MBTL2, CHADL, MAGI1, XKR6, ELAVL2 [4]. We selected up to five results based -log_10_(*p*-value) for with *p* was significant (*p* < 0.05). | |
| --- | --- | --- |
| **Results** | **Schizophrenia (MSigDB hallmark 2020)** | |
|  | Name | -log_10_(*p*-value) |
|  | Bile acid metabolism | 1.307 |
|  | **Depression (MSigDB hallmark 2020)** | |
|  | Name | -log_10_(*p*-value) |
|  | TGF-beta signaling | 1.533 |
|  | **Depression (COVID-19 related gene sets 2021)** | |
|  | Name | -log_10_(*p*-value) |
|  | SARS coronavirus P2 envelope protein from Virus-Host PPI P-HIPSTer 2020 | 2.115 |
|  | SARS coronavirus protein E (gene: E) from Virus-Host PPI P-HIPSTer 2020 | 2.115 |
|  | SARS coronavirus nsp4-pp1a/pp1ab (gene: orf1ab) from Virus-Host PPI P-HIPSTer 2020 | 2.057 |
|  | Top 500 down genes for SARS-CoV-2 infection Day 7 in ferret nasal turbinates from GSE160824 | 1.815 |
|  | SARS coronavirus nucleocapsid protein (gene: N) from Virus-Host PPI P-HIPSTer 2020 | 1.800 |
|  | **Depression (WiKi pathway 2023 Human)** | |
|  | Name | -log_10_(*p*-value) |
|  | SARS-CoV-2 Innate Immunity Evasion And Cell Specific Immune Response WP5039 | 3.238 |
|  | SARS-CoV-2 Altering Angiogenesis Via NRP1 WP5065 | 2.561 |
|  | Hepatitis B Infection WP4666 | 2.531 |
|  | Activation of NLRP3 Inflammasome By SARS-CoV-2 WP4876 | 2.415 |
|  | SARS-CoV-2 B.1.1.7 Variant Antagonises Innate Immune Activation WP5116 | 2.306 |
|  | **Neuroticism (KEGG 2021 Human)** |  |
|  | Name | -log_10_(*p*-value) |
|  | Rap1 signaling pathway | 3.520 |
|  | Neuroactive ligand-receptor interaction | 1.700 |
|  | Ether lipid metabolism | 1.503 |
|  | Cocaine addiction | 1.503 |
| **References** | 1. Chen EY, Tan CM, Kou Y, Duan Q, Wang Z, Meirelles GV, et al. Enrichr: interactive and collaborative HTML5 gene list enrichment analysis tool. BMC Bioinformatics. 2013;14:128.  2. Wu Y, Zhang CY, Wang L, Li Y, Xiao X. Genetic Insights of Schizophrenia via Single Cell RNA-Sequencing Analyses. Schizophr Bull. 2023;49(4):914-22.  3. Levey DF, Stein MB, Wendt FR, Pathak GA, Zhou H, Aslan M, et al. Bi-ancestral depression GWAS in the Million Veteran Program and meta-analysis in >1.2 million individuals highlight new therapeutic directions. Nat Neurosci. 2021;24(7):954-63.  4. Nagel M, Jansen PR, Stringer S, Watanabe K, de Leeuw CA, Bryois J, et al. Meta-analysis of genome-wide association studies for neuroticism in 449,484 individuals identifies novel genetic loci and pathways. Nat Genet. 2018;50(7):920-7. | |
